# Psychoeducation Program on Strategies for Coping with Stress in Patients with Temporomandibular Joint Dysfunction

**DOI:** 10.1155/2014/678169

**Published:** 2014-08-13

**Authors:** Joanna Biegańska, M. Pihut

**Affiliations:** ^1^Department of Medical Psychology, Chair of Psychiatry, Jagiellonian University, Medical Collage, 21a Kopernika Street, 31-501 Krakow, Poland; ^2^Department of Dental Prosthetics, Consulting Room of Temporomandibular Joint Dysfunction, Jagiellonian University, Medical Collage, 4 Montelupich Street, 31-155 Krakow, Poland

## Abstract

Lack of educational projects in the available literature was an inspiration to develop a psychoeducational program. The objective was to provide patients with basic information on the contribution of stressors in the occurrence of temporomandibular joint dysfunction and educate on methods for coping with stress most commonly used in psychology. In the course of three meetings, patients are familiarised with the issue of experienced stress as a potential source of psychosomatic illnesses (in particular, temporomandibular joint dysfunction). Preliminary patients' opinions, expressed through self-report methods, indicate significant usefulness of the developed psychoeducational program for the process of treatment and the quality of patients' lives.

## 1. Introduction

Stress, as indicated by numerous data in the literature, is one of the main etiological factors of functional disorders of the masticatory system [1–11]. More than half of this group of patients experiences high-frequency stress, that is, the number of life events having features of difficult events [2], and even extremely traumatic events. Authors of available publications indicate that this may be the group that did not develop the appropriate skills to cope with stress at a physiological level. In the future, PTSD (posttraumatic stress disorder) symptoms may develop in some of these patients [12–16].

**Figure 1:**
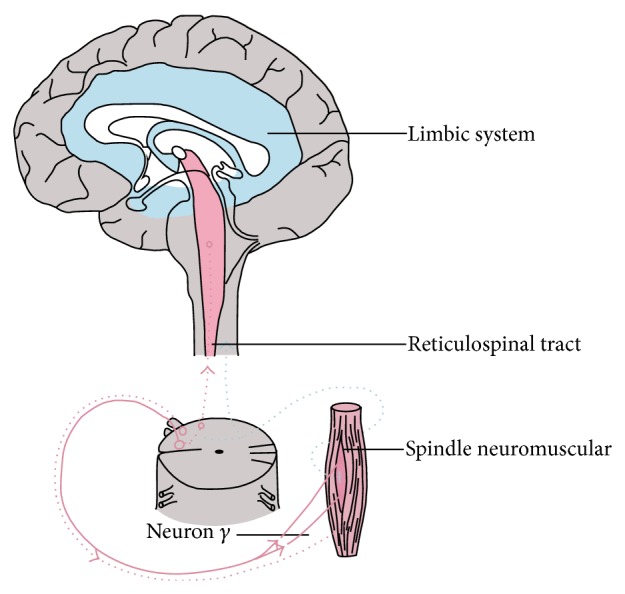
Wiring diagram of the limbic system, gamma loop, and chewing muscle.

**Figure 2:**
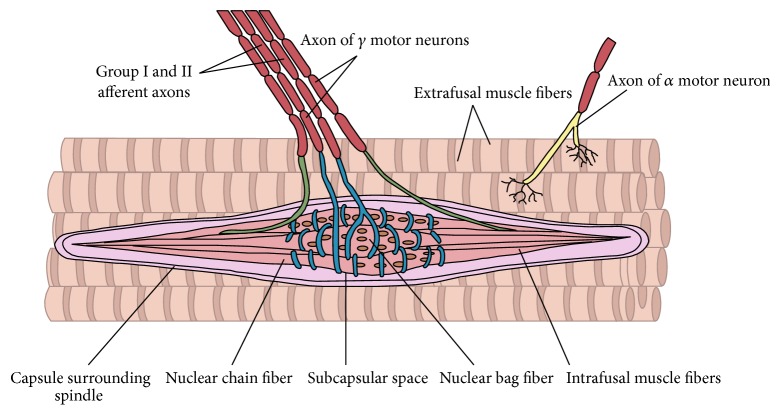
Sketch of afferent and *γ* motor neurons connection with neuromuscular spindle.

The results of neuroimaging studies, mainly using magnetic resonance imaging, have provided valuable information regarding the role of experienced stress in the formation of parafunctions, consequently leading to persistent morphological and functional disorders of the masticatory system [17–21]. Information about stressors is transmitted by the corticobulbar tract to the brainstem structures which, in turn, activates the neurotransmission in the central pontospinal structure of the reticular formation, as a result stimulating the *γ* loop. *γ* cell bodies are placed in anterior horns of the spinal cord, and their fibres reach muscle sensory receptors-neuromuscular spindle, regulating excitability of the latter [22–29] (Figures 1 and 2). Hence, the release of strong emotions accompanying experiencing stress results in the activation of mandibular adductor muscles' activity. Prolonged psychophysical tension contributes to excessive activity of the mandibular adductor muscles, especially superior lateral pterygoid muscle, which has its attachment, among others, in the area of the front part of the articular disc. Its pathological displacement results in lack of coordination of the articular head and disc during the mandible movements, causing acoustic symptoms in the form of glitches. Negative effects of excessive muscle tension triggered under the influence of psychological stress include damage to dental hard tissues in the form of cracks in the enamel of clinical crowns, emergence of wedge-shaped lesions, or pathological tooth wear. Moreover, there are also pathological changes in periodontal tissues and uncontrolled bone atrophy, along with damage to the structures of temporomandibular joints and masticatory muscle overload and growth.

The main purpose of the paper is to describe the psychoeducation program dedicated to patients with temporomandibular joint dysfunction. It will also provide information on the impact of stress on the development of the illness, educating on how to cope with stress and increasing the sense of control over their emotional response.

## 2. The Role of Stress in the Temporomandibular Joint Dysfunction

The long-term impact of the stressor leads to a state of severe mental and physical tension [30, 31] that is relieved by movement reactions of parafunctions nature, such as bruxism [32, 33]. Parafunctional habits of masticatory system muscles, with and without associated chronic pain, long-term treatment requiring numerous visits to specialists, rehabilitation exercises, as well as changes of habits are the source of secondary stress for the patient [34–38]. Stress experienced in such a way, together with other daily stressors with which the patients cope in an ineffective way, intensifies the pain. The effect is further intensified by potentially comorbid sleep disorders [39, 40]. All of these factors significantly reduce the quality of life for patients, negatively affecting their motivation to continue the functional disorders of the masticatory system therapy.

The term “coping” can be viewed in two ways: through the collection of relatively fixed characteristics of the individual (so-called coping styles) and in relation to activities undertaken by him/her in a particularly difficult situation (so-called coping strategies) [41, 42]. Coping, according to the firmly established psychology theory created by Lazarus and Folkman [43], performs two basic functions: (1) task (problem-oriented, instrumental), aiming at improving the unfavourable relations of situational requirements to the individual's abilities, and (2) being associated with emotional self-regulation, oriented to reduce the unpleasant tension and to ease negative emotional states. Studies indicate that patients with temporomandibular joint dysfunction are a group that significantly and more frequently uses nonadaptive strategies for coping with stress, which is general avoidance or wishful thinking [4, 43]. At the same time, in this particular group of patients, the negative impact of such strategies on physical and mental wellbeing was confirmed [44]. Subjects only partially [45, 46] maintain consistency between diagnosed coping style and the content of chosen strategies, or this phenomenon does not occur at all [47]. It may indicate certain dynamism of coping, as well as its flexibility; we can, therefore, expect the undertaking of different activities, depending on the actual characteristics of a person (including his/her knowledge and well-being) and the characteristics of the situation.

At the same time, the main characteristics of the stressor are what significantly differentiate the range of coping strategies chosen by the individual, especially the possibility of controlling it [48]. This control is determined by the ability to control the occurrence, course, and consequences of a particular stressor [49]. For example, chronic stress related to the experience of pain caused by the presence of dysfunctions of the stomatognathic system is, for the patient, largely difficult to control. This is the case until the patient is covered by the specialist's care and learns how to diminish the discomfort or prevent the recurrence of symptoms and gain “control” over his/her own illness. When trying to maintain control over the experienced stress situation, the patient is more likely to activate instrumental strategies, aiming at coping with the difficulty. Without such a control, the ability to activate a strategy focused on the regulation of experienced emotions is important.

## 3. Possibilities for Modification of the Style of Coping with Stress through Psychoeducational Interventions

Following Lazarus and Folkman's assumptions [43] applied to an interactive model of coping with stress, any controlled modification of knowledge and competence of the individual realistically makes it possible for her (to some individually limited degree) to influence the process of coping with stress. In practice, it means that the strategies and styles of coping with stress used by the patient may undergo favourable changes under the influence of deliberate, individually customized psychoeducational activities.

Psychoeducation is defined as a form of psychological help combining psychotherapy with education [50, s.206]. As such, it is inscribed in the holistic model of care for the patient based on the development of specific competencies in the patient.

Meta-analyses examining the impact of psychoeducation on the reduction of stress revealed that shorter psychoeducational interventions, comprising several meetings, give particularly favourable results [51, 52]. This is especially important in the case of psychoeducational intervention designed as multimodal, that is, referring to different techniques within a single program. It is different from targeting one specific way of stress reduction, such as the ability to diminish the effects of stress through specific relaxation techniques; in such cases it is reasonable to schedule more meetings.

## 4. Materials and Description of the Psychoeducational Program

### 4.1. Meeting Purpose

The aim of the original psychoeducational program developed in the Consulting Room of Dysfunction of the Masticatory System, Department of Dental Prosthodontics, Jagiellonian University Medical College in Krakow, is to provide patients with basic information about the stressor contribution in the symptoms development of temporomandibular joint dysfunction and education on ways of coping with stress. Moreover, the purpose is to strengthen the sense of control over emotional reactions of patients by choosing the most favourable method of coping with stress for the specific patient. At the same time, the program is a response to the request to enable patients to widen the sense of affecting stressors—not only the disease, but also all others, which may be experienced. This is a particularly important goal of functional disorders of the masticatory system therapy, in which the stress factor is not only somatic and the mental result of experienced illness, but also an important etiologic and dysfunction-supporting factor.

### 4.2. Participants

The participants are Consulting Room of Temporomandibular Joint Dysfunction patients, 18 years old and above, and interested in the program. The only exclusion criterion is the presence of an organic mental disorder or any somatic illness, which prevents them from effective participation at meetings. They are mostly obtained from their dentists, who briefly inform them about the possibility of participating in the program. Already, a few patients refused to participate in the program, indicating some logistical difficulties (access to the meeting place, busy work schedule). However, all of them expressed their keen interest in the issue. What seems interesting is that few participants underwent psychotherapy. None of the participants has ever participated in any psychoeducational program or been instructed to cope with stress.

### 4.3. Content and Methods Used

In the course of three meetings with a psychologist, patients gain knowledge on stress and its impact on their bodies. They also discover their style of coping with stress, and, consequently, have the opportunity to consider, together with a psychologist, which ways of coping with stress (customized to their individual style for coping with difficult situations) may be effective for them. In each case, these meetings are customized to the mental and physical capacity and resources presented by the patient. Each meeting, takes from 45 to 90 minutes, depending on patients' needs, capabilities, and knowledge.

During the first meeting, basic knowledge on the mechanisms of the stress response and psychosomatic in the stomatognathic system is transferred to patients. In many cases, patients independently come to the conclusion that such situations are composed of both the positive and negative. With the help of Holmes and Rahe's Social Readjustment Rating Scale (SRRS) [53], patients have the opportunity to estimate the cumulative stress to which they were recently exposed. Using this scale, life stress is measured in Life Changes Units (LCU). The highest value is assigned to the most stressful events. All the data obtained in the questionnaire are analysed quantitatively. The study conducted by the authors of the scale shows an interesting relationship between the amount of stressors and the likelihood of developing a serious illness. For values exceeding 300, it is almost 80%. During the same meeting, patients are familiarized with the characteristics of personality types, which may favour the occurrence of difficulties in the fight against stress. Personality may be related to stress resistance, as it may determine the quantity and quality of positive social and family relationships, which can further ease the impact of stress and reduce the number of incidences and premature deaths [54]. In contrast to introverts, people who are open and oriented toward contacts with other people (extroverts) may have more people in their environment who are ready to offer help in difficult situations, in this way weakening the negative effects of stress. So-called neuroticism—a tendency to worry and anxiety, especially when connected with a low level of extraversion—is a feature which lowers the overall stress resistance. Further work, during the first meeting with a psychologist, focuses on making patients aware of the prevalence of stressors and difficulties in adequately coping with them, giving special attention to the group of patients with dysfunctions in the stomatognathic system. At this stage patients, during a short lecture, are familiarized with the issue of stress as an etiologic factor of experienced temporomandibular joint dysfunction. Explanations concern a two-way relationship of stress and health [55], pointing to the basic physiological and psychological processes: emotions, behaviours accompanying stress, and coping.

In the last part of the first meeting, the psychologist explains harmful and unfavourable changes occurring in the organism under the influence of stressors, on the example of the mechanisms of developing functional disorders of the masticatory system. Another issue discussed at the first meeting is presenting the possibilities of analysing and avoiding situations that generate experienced stress and enriching types of strategies for coping with stress using potentially the most beneficial methods for the particular patient.

The second psychoeducational meeting begins with the completing of a Brief-Cope survey (Coping Inventory for Stressful Situations, Polish adaptation: Z. Juczyński) on the scale of coping with stress by the patient. The questionnaire consists of 28 statements included in the 14 strategies (two statements for each strategy). It is used to measure dispositional coping and the assessment of the typical ways of responding and emotions in difficult situations. The advantage of this questionnaire is the possibility of quickly summarizing the results and providing patients with feedback during the current meeting. Although the main method of data obtained in the questionnaire analysing was quantitative, it was supplemented by participant statements, analysed qualitatively.

Moreover, possibilities are also discussed to modify the unconstructive behaviour within the frame of strategy for coping with stress preferred by the patient. It occurs during the motivating interview, which is designed to strengthen a patient's involvement in making significant life changes and facilitate the discovery of intrinsic motivation for changes. Motivating interview is a style of work, based on partnership cooperation with a specialist (in this case a psychologist), with the patient striving to transform his/her attitude into a pro-health perspective [56].

The purpose of the third, and last, meeting is to familiarize patients with constructive techniques for coping with stress, consistent with their individual predispositions. They are proposed on the basis of the subjective examination results and the Brief-Cope questionnaire. They include both complex techniques, such as physical self-regulating techniques, relaxation techniques (e.g., Jacobson progressive relaxation training), or self-hypnosis, as well as simple methods based on instruction in physiological breathing in stressful situations. It is a meeting during which the patient is able to practically experience the basics of techniques, which he considered interesting or useful. Moreover, the meeting highlights a significant role of contacts with relatives, regular physical exercise or sleep hygiene, factors which (as revealed in studies) are an important source of dysfunction of stomatognathic system.

## 5. Results and Discussion

The paper provides a description of the psychoeducational program for patients with temporomandibular joint dysfunction, which may be investigated as replenishment treatment. As a result the exact research, supported with objective measurements, is essential.

Initial patients' opinions indicate the usefulness of psychoeducation in the field of treating functional disorders of the masticatory system and present an important impetus to continue psychoeducational meetings. Patients emphasize the benefits of analyzing their current psychophysical situation, expressing satisfaction of the new knowledge, which is used in everyday life, and they are interested in the possibility of continuing education in specific techniques pointing to stress reduction. Moreover, meetings show that skillfully directed patients demonstrate high creativity in designing techniques, which are appropriate for them. Sometimes patients communicate their knowledge to people within their immediate environment, thereby encouraging collective use of previously acquired knowledge.

The proposition is not without some limitations. Firstly, a significant amount of content to pass on to patients in a small window of time requires considerable and efficient cooperation. Secondly, the construction stage of the program did not include physician participation in meetings and completing the psychological data with some physiological or medical knowledge, strengthening the power and reliability of the transmission. Finally, the personal factor should also be noted; the implementation of the program would require the creation of a consistent training for psychologists having to conduct the meetings with patients. The temporomandibular joint dysfunction issue is still not very popular among the representatives of the profession (at least in Poland).

Developed psychoeducational program is the second proposal of patients' education in techniques for coping with stress. A psychoeducational program project for patients with dysfunction of the temporomandibular joints, created at the Eastman Dental Institute, University College London, seems to produce equally positive results [57]. Psychoeducational interventions used in the project were related to two aspects of psychoemotional functioning of patients and were aimed at developing a sense of control and self-efficacy, as well as self-relaxation (in two independent groups). The main fundamental difference between these two programs is their form. In the case of the London proposal, it was focused on training in the field of a particular method; thus, patients had the opportunity to be familiarized with this technique for coping with stress, but they did not obtain the knowledge on the mechanisms which justify the importance of undertaken psychoeducational impacts. In the case of this program, the techniques which were a part of psychoeducation were selected with regard to individual characteristics of the patient. In the case of the London program, it was not driven by psychoemotional characteristics of individuals. Comparing the effectiveness of training in order to create the optimal method of impacting patients with temporomandibular joint dysfunction would be an interesting proposition. However, it seems to the authors that the psychological impact on this group of patients should proceed in two stages, combining the proposed psychoeducational program with training in specific techniques for coping with stress.
